# Techno-Economic Assessment of Two Process Routes for Lignin-Derived Alkylphenols and Aromatic Hydrocarbons

**DOI:** 10.1021/acsomega.5c08334

**Published:** 2026-02-06

**Authors:** Aristide Giuliano, Aniello Di Giacomo, Isabella De Bari, Diego Barletta

**Affiliations:** † 18114 ENEA − Italian National Agency for New Technologies, Energy and Sustainable Economic Development, ENEA Division Bioenergy, Biorefinery and Green Chemistry, S.S. 106 Ionica Km 419 + 500, Rotondella, MT 75026, Italy; ‡ Department of Industrial Engineering, 19028 University of Salerno, Via Giovanni Paolo II 132, Fisciano, SA I-84084, Italy

## Abstract

Lignin valorization processes are crucial for enhancing the economic feasibility of lignocellulosic biorefineries and improving waste management in the pulp and paper industries. This study performed a detailed techno-economic evaluation comparing two alternative lignin conversion pathways, focusing on the production of aromatics and alkylphenol: HydroDeOxygenation (HDO) and HydroDeOxygenation are preceded by HydroThermal Liquefaction (HDO + HTL). The analysis revealed that the integrated HDO + HTL is technically and economically superior, since it provides higher alkylphenol yield (30–35% by weight) and requires significantly less hydrogen, consuming only 22–28 kg of H_2_ per ton of lignin. As a direct consequence of the superior yield and lower reagent consumption, the minimum selling price of alkylphenols via HDO + HTL is less than half of that obtained via HDO (1.52 compared to 3.47 EURO/kg, respectively). A comprehensive sensitivity analysis demonstrated that the lignin market price has the greatest impact on the minimum selling price of alkylphenols, resulting in variations of more than EURO 1/kg_AP_. Even under the most favorable economic conditions for direct HDO (low hydrogen cost and free lignin), the HTL + HDO configuration remained the most economically convenient process solution. The results affirm that coupling HTL with HDO maximizes product yields and minimizes costs, securing profitability for lignin valorization

## Graphical abstract

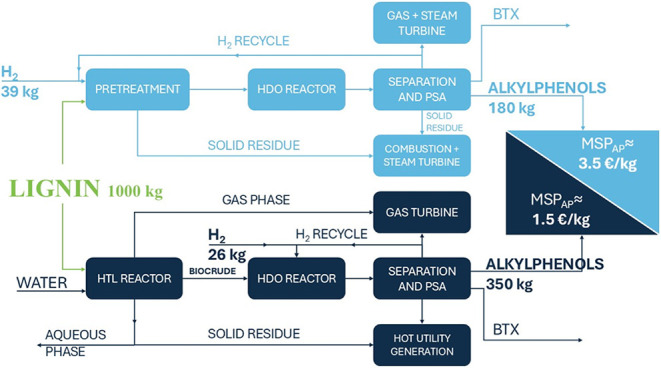

## 1 Introduction

Biorefineries convert biomass into fuels, high-added value chemicals, and energy in a sustainable way.^1^ Lignocellulosic biomass is formed by three main components: cellulose, hemicellulose, and lignin. The valorization of the first two is effectively achieved at the industrial scale.^2^ Lignin is a polymer with a variable, irregular structure and with a mass fraction in biomass ranging from 16% (as in miscanthus) to more than 26% (for example, in eucalyptus).^3^ Lignin is currently underutilized since it is usually only thermally valorized.^4^ Nevertheless, lignin is the largest renewable source of aromatic biopolymers on the planet and has a lower oxygen content concerning cellulose and hemicellulose, which makes it more suitable for the conversion into high-energy-density chemical products, like biofuels.^5^ For future perspectives, a crucial step forward could rely on the chemical conversion of lignin to improve the techno-economic feasibility of both new and existing biorefineries.^6^ Many options have been studied for this goal,^7^ but the chemical recalcitrance of the feedstock can be very limiting.

A possible pathway to obtain high added-value chemicals from lignin is based on the hydrodeoxygenation (HDO).^8^ By using pressurized H_2_ in the presence of an appropriate catalyst, lignin can be depolymerized while chemically bonded oxygen can be removed in the form of H_2_O (or as CO/CO_2_ in the worst-case scenario): aromatic ring saturation can also take place. Under these conditions, lignin reactivity is due to the presence of two kinds of bonds (β-O-4 and α-O-4),^9^ which share a low bond dissociation energy.^10,11^ Recently, another lignin depolymerization route consisting of hydrothermal liquefaction (HTL) has generated a lot of interest. This is a thermochemical process able to convert biomass or other feedstock into a liquid fraction, usually called biocrude or bio-oil, using water in the supercritical state (*T* ≥ 374.1 °C and *P* ≥ 221 bar) or in the subcritical state. However, according to Castello et al.,^12^ after an HTL step, further biocrude upgrading is required to improve the product properties (for example, lowering the oxygen content for a possible biofuel application) and its economic value. Modeling liquefaction processes is hindered by the difficulties in describing the molecular structure of heterogeneous lignin materials. As a result, the overwhelming majority of these studies were conducted assuming so-called model compounds or equivalent components.^13^ Laskar et al.^14^ stated that to understand the key principles involved in lignin catalytic depolymerization, monomeric equivalent components, such as phenols, anisoles, and their substituted derivatives, have to be considered. Some studies reported the conversion of lignin-derived monomers by HDO into alkylphenols, valuable intermediates, and green solvents.

Process systems design is crucial to demonstrating the feasibility of lignin valorization in biorefineries and waste management plants. Process simulations often treat the lignin reaction section as a “black box” based on experimental data. Recent studies by Acevedo-García et al.^22^ focused on lignin biorefineries producing propylene and ethylene, showing reduced climate change impact. Weyand et al.^23^ analyzed lignin catalytic depolymerization for aviation fuel, noting high carbon and energy efficiency, but lignin was the highest cost and environmental impact contributor. Ahire et al.^24^ studied lignin for resin production. Bagnato et al.^25^ analyzed a hydropyrolysis plant, highlighting the need to improve bio-oil yield for economic viability. Shen et al.^26^ conducted a techno-economic analysis of direct lignin HDO for jet fuel, modeling lignin as vanillin. Vural Gursel et al.^27^ compared lignin direct HDO with pyrolysis and hydrothermal upgrading. Bbosa et al.^28^ conducted a techno-economic analysis of standalone HTL in a biorefinery context. Lignin HTL often requires a preceding hydrogenation step, as the biocrude needs upgrading. Tito et al.^29,30^ examined HTL and hydroupgrading for biofuel, achieving a minimum selling price (MSP) of 1.27 EURO/kg. Both HTL and HDO convert lignin by using high temperatures, pressures, and/or catalysts but face challenges in product selectivity and economics. Combining HTL and HDO aims to maximize yields and improve techno-economic viability. The availability of more detailed process simulation models would significantly improve the accuracy of evaluating the techno-economic performance of plants with chemical valorization of lignin.

This study aims to improve the techno-economic evaluation of lignin valorization plants significantly by developing rigorous simulation models for two promising routes: direct lignin HDO and the combined HTL and HDO process for the coproduction of an alkylphenol (cresol) and aromatic hydrocarbons (benzene, toluene, and xylene). The novelty of this work lies in a detailed and comparative techno-economic assessment built upon accurately modeled reaction sections that integrate realistic kinetic data from the existing literature. Through precise sensitivity analyses within these simulations, we will identify optimal process conditions for each configuration including HTL residence time and inlet temperature, HDO operating pressure, and the necessary hydrogen consumption. The best-case scenarios for both technical performance (e.g., yield of desired products) and economic viability (costs and revenues) will then be rigorously compared. Finally, the minimum selling price of the target alkylphenol will be more precisely calculated and used as a key metric to determine the most promising process.

## 2 Methods

### 2.1 Process Design and Modeling

#### 2.1.1 Lignin Modeling

Kraft pine lignin, free of hemicellulosic material and with a lignin content of 97 wt % on a dry basis, is the considered feedstock of this study. Its complex polymeric structure is modeled as a mixture of oligomers, made of the most common structural units such as *p*-hydroxyphenyl (H), guaiacyl (G), and syringyl (S) units. In this study, C_38_H_44_O_14_ and C_30_H_36_O_11_ oligomers were chosen, since they are characterized by a percentage of C–O–C (β-O-4) and C–C (5–5′ and β-1) chemical bonds very similar to that of the Kraft pine lignin.^31^ These oligomers were implemented in the property model section of the Aspen Plus 12.1 process simulation software by providing the chemical structure (reported in Figure S1 in the Supporting Information) and temperature-dependent thermophysical properties taken from Wooley and Putsche,^15,16^ and Azad et al.^32,33^ Further details are reported in the Supporting Information.^34,35^

#### 2.1.2 Lignin Depolymerization Reactions

A complex reaction network was assumed for the Hydrodeoxygenation (HDO) of lignin oligomers (Figure 1). This network includes the depolymerization to create a wide variety of chemical components: Phenolics (Phenol, Guaiacol, Syringol, Catechol, Cresol), Aromatic Hydrocarbons (BTX-type) (Benzene, Toluene, Xylene), Cycloalkanes and Alcohols (Cyclohexane, Methyl Cyclohexane, Dimethyl Cyclohexane, Cyclohexanol, Ethanol, Propenol); Ethers (Anisole, Dimethoxybenzene), Aldehydes and Acids (Hydroxybenzaldehyde, Vanillin, Syringaldehyde, Acetic Acid, Formic Acid), light gases (Hydrogen, Oxygen, Methane, Carbon Monoxide, Carbon Dioxide) and Water. The final composition of the reactive system component was calculated according to a simplified thermodynamic-based method, the so-called temperature approach to the equilibrium method, which is commonly used to predict real conversion values not corresponding to a complete chemical equilibrium state.

**Figure 1:**
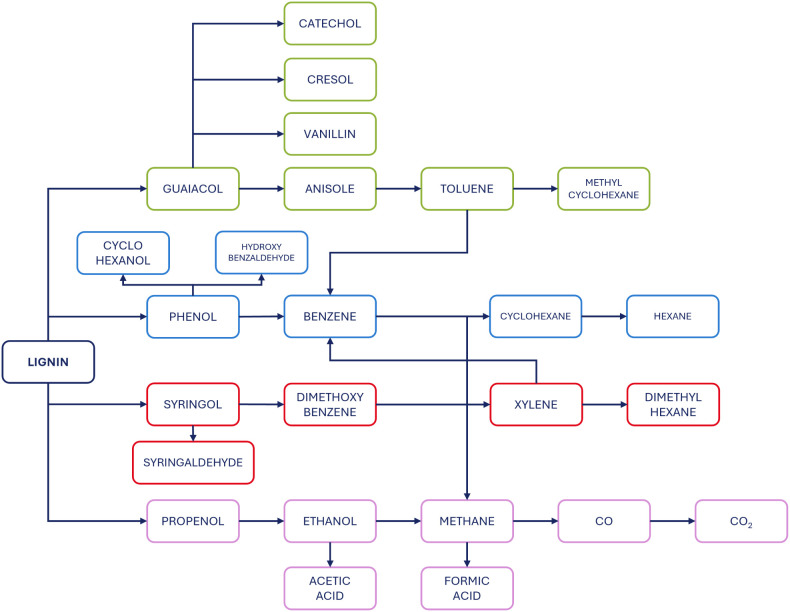
Assumed reaction network of the lignin Hydrodeoxygenation.

This implies that each reaction *i* is assumed to reach chemical equilibrium at a temperature value, deviating by Δ*T_i_* from the real reaction temperature of 350 °C. The set of Δ*T_i_* values was obtained by minimizing the mean squared error between the predicted and the experimental values of the gas stream composition leaving the reactor with the catalyst (S-NiMo/MgO-La_2_O_3_) reported by Kumar et al.^31^ Further details can be found in Table S3 in the Supporting Information.

The complex lignin depolymerization network by Hydrothermal Liquefaction (HTL), generating a large spectrum of products (methoxy-phenols, phenols, catechols, etc.) is modeled by adapting the lumped kinetic model proposed by Forchheim et al.,^17–21^ They introduced two intermediates, LD1 and LD2: the first lump embraces somewhat soluble lignin, with a significantly reduced molecular weight but is still chemically reactive, while the second lump consists of completely stable, partially water-soluble depolymerized lignin, which is not reactive anymore. This network model is reported in detail in Figure S2 and in Table S4 of the Supporting Information.

##### 2.1.2.1 Process Flowsheet Design

The lignin feedstock flow rate was assumed 9350 kg/h, based on the lignin output stream of the existing lignocellulosic biorefinery, located in Crescentino (Vercelli, Italy).^36^

#### 2.1.3 Lignin Direct HDO Section

The process flowsheet of the direct HDO configuration is shown in Figure 2a. Lignin and hydrogen, provided by a fresh hydrogen makeup stream and a recycle stream, are mixed and heated to the reaction temperature of 350 °C of the HDO reactor.^31^ The reactor volume was estimated by assuming a 4 h residence time. Since it is not possible to evaluate the effect of catalyst regeneration and deactivation, fresh catalyst was always considered. In particular, the required fresh catalyst amount was assumed to be 5% by weight of the lignin inventory in the reactor.

**Figure 2:**
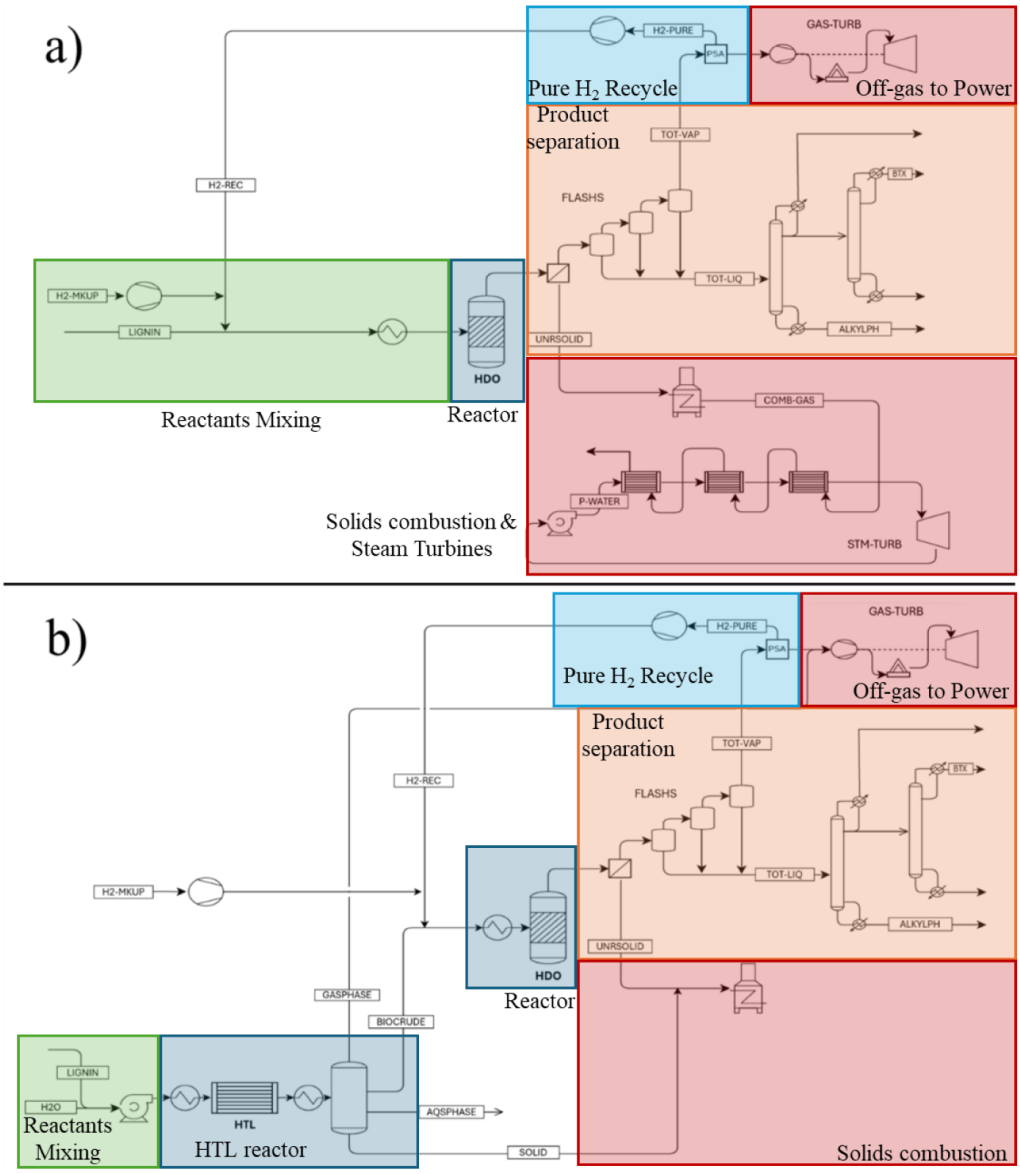
Process flow diagram for a) the direct HDO and b) the HTL + HDO processes.

The hydrogen-to-carbon H/C ratio in the feed (between 4 and 6) and the reactor pressure (between 50 and 100 bar) are the principal process degrees of freedom investigated in this study for the HDO reactor performance and economic optimization of the overall process.

#### 2.1.4 Lignin HTL + HDO Section

The HTL + HDO process flowsheet is represented in Figure 2b. The lignin feedstock stream is mixed with water to form a slurry, which is then pumped and preheated to obtain high-pressure boiling feedwater for the subsequent HTL tubular reactor (ID = 3 m). The HTL depolymerization reactions are mildly endothermic, and given the high heat capacity of water, an adiabatic reactor was assumed; therefore, the heat transfer effects are not considered.

The first separation of the reactor effluent is carried out by gravity in a decanter^38^ to obtain four streams: a biocrude stream, made of desired lignin oligomers and other organic products (methoxycatechols, catechols, phenols); a gaseous phase stream, mainly composed of H_2_, CO, CH_4_ and CO_2_; an unreacted lignin solid phase stream; a water-rich stream with a limited fraction of organic lignin depolymerization products.

Water-to-lignin ratio in the feed^17,39^ (varied between 5, 7.5, and 10), reactor temperature (varied in the range 320–370 °C), and residence time (up to 120 min) are the principal process degrees of freedom investigated in this study for the HTL reactor performance and the economic optimization of the overall process.

The valuable biocrude stream is fed to an HDO reactor to be upgraded^37^ further by depolymerization of the reactive lignin oligomers LD1.

#### 2.1.5 Alkylphenols and Aromatics Purification and Hydrogen Recovery Section

For both configurations, the HDO reactor effluent vapor stream at 350 °C is sent to a filter to remove the unreacted solid phase. Afterward, a sequence of partial condenser units allows the fractionation into liquid streams, containing the high-value products alkylphenol (cresol) and BTX, and vapor streams, made of incondensable gas mixtures with about 10% of hydrogen. Separation and purification of the liquid streams are obtained by two distillation columns: alkylphenols with a purity ≥98% are recovered from the bottom of the first column, while aromatic hydrocarbons (BTX), with a purity ≥95% are recovered in the distillate of the second column. The bottom aqueous stream containing organic compounds undergoes wastewater treatment. Hydrogen in the vapor stream resulting from the partial condensation train is recovered and purified by pressure swing adsorption (PSA) and compressed and recycled to the HDO reactor to reduce the demand for fresh hydrogen, which significantly affects the plant’s economic feasibility. The remaining gas stream is sent to the energy recovery section, which is modeled according to the simplified approach by Giuliano et al.^40^

#### 2.1.6 Combined Heat and Power Production Section

A combined heat and power production system with gas and steam turbines is included in the plant to thermally valorize off-gas streams and unconverted solid residue stream to minimize the expenses for hot utilities and electricity.^41^ For this reason, both direct HDO and HTL + HDO process configurations need to be completed with a combined heat and power production system. The solid residue is used in both cases to satisfy the plant’s hot utility need, and a gas turbine system can thermally valorize the off-gas streams to produce electric energy. In particular, a steam Rankine cycle can be used only in the case of the direct HDO configuration due to a significant amount of unconverted solid residues to thermally convert.

Heat integration was possible and verified by the Pinch technology approach. In particular, the Grand Composite Curves, obtained by solving the problem table and reported in Figure S5 of the Supporting Information, indicated that external hot utilities are not needed for both the HDO process and the HDO + HTL process, as it happens in threshold problems. A simplified approach was used to couple process hot streams and cold streams. Cold streams with a final temperature around 350 °C were heated by flue gases; cold streams with a final temperature up to 200 °C were heated by high-pressure steam, generated by flue gases in excess, or by hot process streams available at more than 250 °C; cold streams with a final temperature below 50 °C were heated by hot process streams with temperatures above 100 °C.

Surplus electricity generated in the direct HDO process is exploited by an electrolyzer producing hydrogen from water to reduce the operating expenditure.^42–47^

### 2.2 Economic Analysis

An economic analysis was performed to compare the two processes under scrutiny, assumed as grassroots plants. Capital costs (CAPEX) and operating costs (OPEX) were computed for both direct HDO and HTL + HDO process configurations. Capital costs (CAPEX) for the equipment were computed using power law correlations as a function of the unit capacity using the values summarized in Table S5 reported in the Supporting Information.

The assumptions made to compute the operating costs (OPEX) are summarized in Table S6 reported in the Supporting Information.

In this study, the minimum selling price (MSP) of the alkylphenols is considered as the main economic performance indicator of the different process configuration. By definition, it is the value of the product selling price that makes the Net Present Value, NPV, equal to zero at the end of the plant’s life (20 years). NPV can be calculated by a discounted cash flow analysis:

1 $$NPV=\overset{n}{\underset{j=0}{\sum }}\frac{CF_{j}}{{\left(1+i\right)}^{j}}=\overset{n}{\underset{j=0}{\sum }}\frac{-f_{j}\cdot TIC+g_{j}\cdot WC+\left(Rev_{j}\left(MSP_{AP}\right)-TAC_{j}\right)\left(1-t\right)+DP\cdot t}{{\left(1+i\right)}^{j}}=0$$

where CF_*j*_ is the annual cash flow for year *j*, *i* is the interest rate (5%),^48^ *n* is the expected number of years of the plant’s life (20 years), TIC is the total capital investment cost, *f*_*j*_ is the fraction of the TIC spent during year *j*, WC is the working capital, *g*_*j*_ is a parameter equal to −1 for *j* = 3 (the final year of the plant’s construction) and to 0 for all other values of *j*, Rev_j_ are the annual revenues, TAC_j_ is the total annual operating cost, *t* is the tax rate, and DP is the depreciation.

However, in the numerous sensitivity analyses performed, the MSP of alkylphenols was calculated by this simplified equation:

2 $$MSP_{AP}=\frac{\left(CC+TAC_{lig\&H2}+TAC_{lab\&main}+TAC_{util}\right)-\left(Rev_{BTX}+Rev_{elect}\right)}{{ṁ}_{AP}}$$

where TAC_lig&H2_ is the total annual cost for raw materials and reactants, lignin and hydrogen, TAC_lab&main_ is the total annual cost for labor and maintenance cost, TAC_util_ is the total annual cost for the utilities, Rev_BTX_ is the revenue for the sales of the byproduct BTX, Rev_elect_ is the revenue for the sales of the electricity, ṁ_AP_ is the mass flow rate of AP, CC is the annualized capital cost computed as the product of the TCI and of an annualization factor, AF, divided by the number of operating hours per year:

3 $$CC=\frac{TCI\cdot AF}{hours/year}=\frac{TCI\cdot \frac{{\left(1+i\right)}^{n}\cdot i}{{\left(1+i\right)}^{n}-1}}{hours/year}$$

A sensitivity analysis on both lignin and hydrogen costs was carried out. Lignin’s average cost was assumed to be 200 EURO/t, and a variation of ±200 EURO/t was considered to span from the worst-case scenario, including high transportation cost, to the best-case scenario, with locally available lignin produced in situ as a waste stream in the same biorefinery. Furthermore, hydrogen’s cost can vary in a wide range depending on its production method and primary energy source.^49^ Upper and lower bounds are given by the cost of green hydrogen, obtained through the electrolysis completely powered by renewable energy, and the cost of gray hydrogen, produced by methane steam reforming, without carbon capture and storage (CCS) systems. Hydrogen’s average cost was assumed to be equal to 4 EURO/kg, which is higher than the price for blue H_2_, produced from fossil fuel but with CCS. The variation chosen is ±2 EURO/kg, to consider the worst scenario close to the current one and the optimistic outlook in which hydrogen cost could decrease due to a surplus production of renewable energy.

## 3 Results

### 3.1 Process Analysis

For the direct HDO process, the reactor residence time was set to a constant value of 4 h, taken from the experimental work of Kumar et al.^31^ As a result, the reactor size and catalyst increased with increasing H/C ratio and decreasing pressure. For the HTL reactor, economic optimization suggested a residence time between 40 and 50 min and the lowest inlet temperature possible. Optimum conditions maximize the production of the lignin reactive intermediate LD1, which further reacts in the HDO to yield the high-added-value main products alkylphenols (AP) and the byproducts benzene, toluene, and xylene (BTX). The maximum yield to LD1 does not correspond to the maximization of the biocrude flow rate. This condition corresponds to a higher yield to LD2 that cannot be converted to AP in the HDO reactor.

The HDO reactor volume and the required catalyst amount for the HTL + HDO configuration were estimated by considering the biocrude liquid volumetric inlet flow rate; as a result, for a larger H/C ratio, the required reactor size and catalyst amount accordingly increase.

The mass yield to alkylphenols, computed on a lignin feed basis, is reported as a function of the H/C ratio in Figure 3a for both process routes. Inspection reveals that the yield achievable by the HTL + HDO configuration is from two to three times that achievable by the direct HDO, depending on the H/C ratio value. The presence of the HTL step can partially depolymerize the lignin feed and hence dramatically improve the HDO reactor performance. Moreover, for the direct HDO, the yield increases with the H/C ratio, while for the HTL + HDO the opposite effect is observed. This difference can be explained by considering the extent of overall lignin depolymerization. In direct HDO, the role of H_2_ is to depolymerize the fed solid lignin and to hydrogenate and deoxygenate intermediate compounds to obtain the final desired products. Conversely, in the HTL + HDO configuration, depolymerization occurs almost entirely in the HTL step; the hydrogen reacting later in the HDO step has only the role of upgrading the biocrude. Choosing an excessively large H_2_ inlet flow rate could then result in obtaining overly promoted HDO reactions, which may use the desired products and convert them into the ultimate hydrogenation products, CH_4_ and H_2_O. For this reason, a low H/C ratio is to be preferred to obtain the maximum alkylphenols yield.

**Figure 3:**
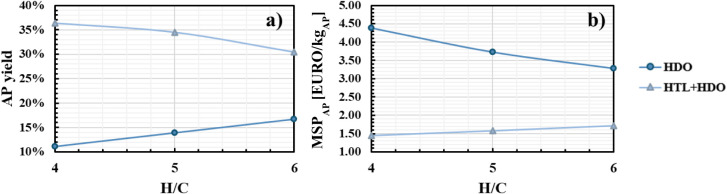
a) Alkylphenols yield; b) Alkylphenols MSP for direct HDO (circles) and HTL + HDO (triangles).

Pressure changes in the range of 50–100 bar appear to have a very limited effect on the yield (less than 5% of the value) for both process pathways. However, it should be considered that this result could be biased by the simplified thermodynamic approach in the HDO reactor modeling, which could not fully take into account the eventual effect of pressure.

On the whole, the maximum yield to AP of about 35% was obtained for HTL + HDO at H/C = 4 and *P* = 100 bar. BTX production is promoted by a high H/C ratio as well, while increasing pressure has a negative effect; the yield value is, however, comparable for the two processes, ranging from 5% to 7% in both cases. Further details concerning the effect of the water-to-lignin ratio (*W*/*L*) on the yield to products of the HTL process step are reported in Figure S3 of the Supporting Information.

The demand for fresh hydrogen for both process configurations is reported in Figure 4. As expected, a larger quantity of hydrogen per ton of lignin is required for higher H/C values in both process configurations, ranging from 26 to 40 kg/t in the direct HDO process and from 20 to 27 kg/t in the HTL + HDO process. The comparison highlights that the direct solid lignin HDO process will always require more H_2_ since the latter is used both to depolymerize and to hydrogenate, even though it produces fewer alkylphenols overall. Differently, in the HTL + HDO configuration, despite a higher yield to AP, an overall lower amount of hydrogen is necessary since hydrogen is not used in the depolymerization taking place in the HTL, but only to upgrade the resulting biocrude stream.

**Figure 4:**
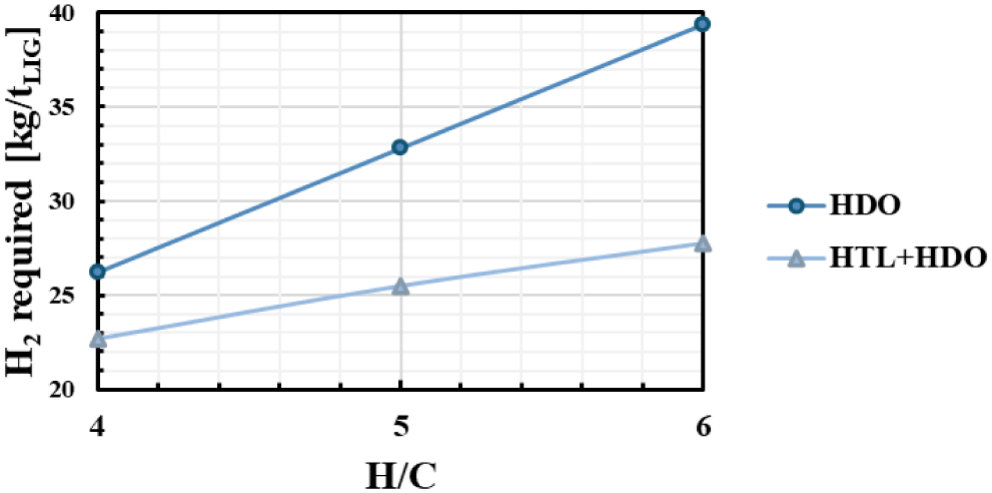
Hydrogen demand per ton of lignin for direct HDO (circles) and HTL + HDO (triangles) configurations.

Sankey diagram representing the overall mass balance for the direct HDO and the HTL + HDO process optimal cases is reported in Figure 5.

**Figure 5:**
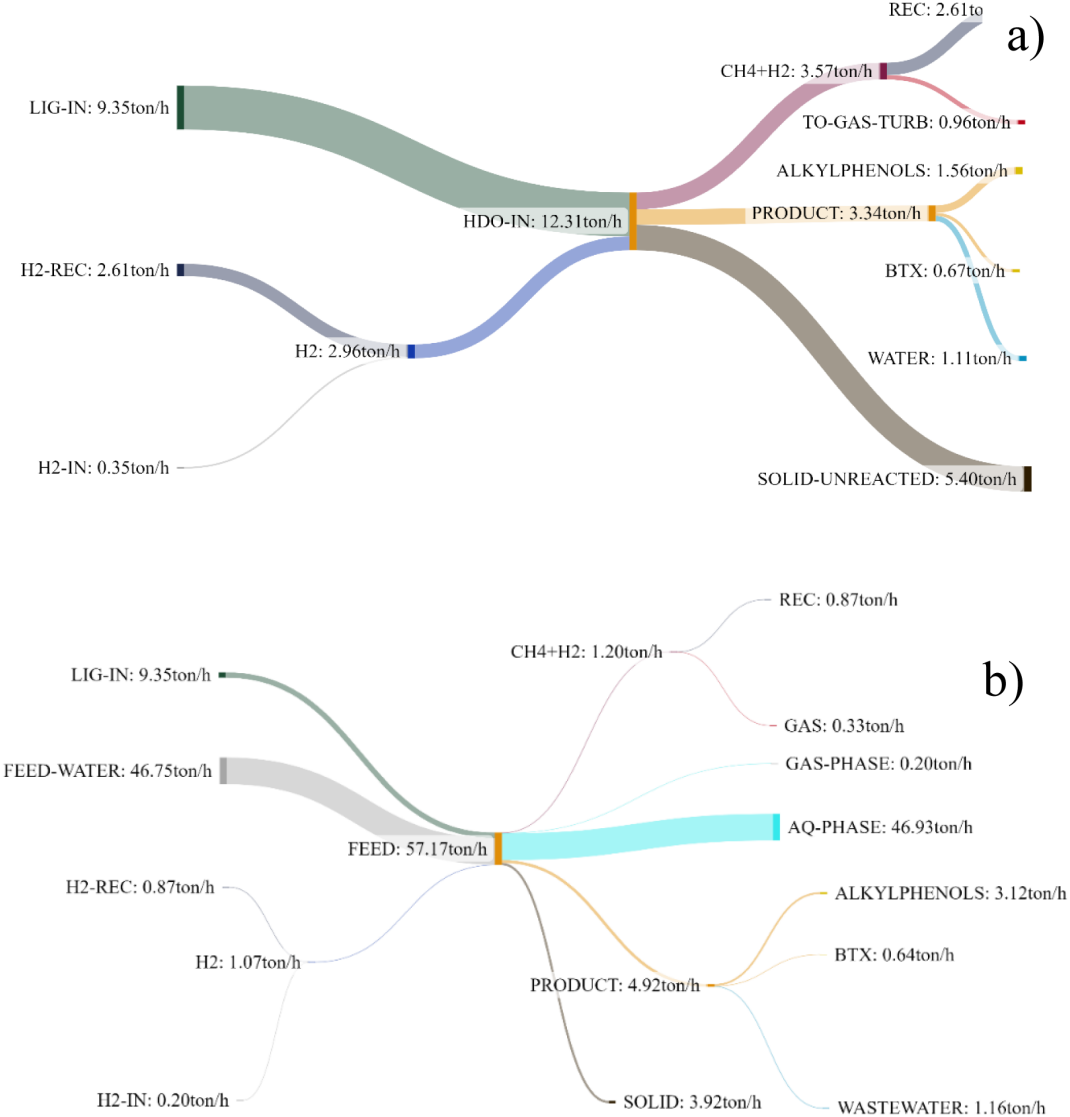
Sankey diagrams representing the mass balance for a) the HDO process and b) the HTL + HDO process optimal cases.

From an energy perspective, it is important to underline that the HTL + HDO configuration with a water-to-lignin ratio larger than 5 is not feasible, since it is not thermally self-sufficient by heat integration. This means that the energy required to heat up the feed cannot be provided by recovering the heat of combustion of the off-gas and the solid residue streams (SOLID + UNRSOLID in Figure 2b). Therefore, *W*/*L* was upper limited to 5 in the results of this work. Hence, both configurations turn out to be thermally self-sufficient, meaning that there is no need to provide external hot utilities generated by fossil sources such as natural gas. The two configurations provide different results in terms of the generated electric energy. The direct HDO process was integrated with three energy generation units (Figure 2a):

- a gas turbine for the process off-gases derived from the PSA unit (a stream rich in CO_2_, CO, CH_4_, and H_2_);
- a steam turbine utilizing the sensible heat of the flue gases from the gas turbine through a Heat Recovery and Steam Generator;
- a steam turbine harnessing heat from a burner and boiler, which burns the residual solid unconverted lignin (UNRSOLID in Figure 2a) directly from the HDO reactor after filtering.

On the other hand, in the HTL + HDO process configuration (Figure 2b), the solid residues from the first reactor (SOLID in Figure 2b), the residual solid unconverted biocrude (UNRSOLID in Figure 2b) and the flue gases derived from the gas turbines were used to thermally sustain the HTL reaction. Consequently, only one gas turbine system was introduced to produce electricity by burning the off-gas stream.

Overall, the electric power produced in the direct HDO case is significantly larger (12–14 MW) than that obtained from the HTL + HDO configuration (up to 1 MW).

This finding plays a significant role in proving the reduced environmental impact of the process.

### 3.2 Economic Assessment

Alkylphenols’ MSP calculated for both process configurations as a function of H/C is reported in Figure 3b. The lowest MSP_AP_ value is equal to 3.47 EURO/kg_AP_ and to 1.52 EURO/kg_AP_ for the direct HDO process and the HTL + HDO configuration, respectively, both at 60 bar. In the second case, optimal conditions for the HTL reactor are obtained for a residence time of 50 min and an inlet temperature of 320 °C. The MSP_AP_ value for the direct HDO process is comparable with the market price of AP reported in the literature (2.51–3.14 EURO/kg),^28^ while the MSP_AP_ for the HTL + HDO process is lower. As a result, the latter configuration appears to offer profit opportunities.

Figures 3b and 4 highlight that the lowest MSPs of the two processes correspond to different hydrogen demand values: in the direct HDO it is 39.5 kg/t of lignin (H/C = 6), while in the HTL + HDO it is 22.5 kg/t of lignin (H/C = 4). In the HTL + HDO process, significantly less hydrogen is required due to the prior depolymerization and partial hydrogenation facilitated by the reactive role of water during the HTL phase. This reduces the hydrogen demand in the subsequent HDO process step. Conversely, in the direct HDO process, the entire sequence of depolymerization and hydrogenation reactions must take place within a single HDO reactor, leading to a substantially higher hydrogen consumption. Of course, this difference affects the operating costs. Henceforth, the economic performances of the optimal cases were compared in further detail. In particular, Figure 6a shows that CAPEX for the HDO process is 30% higher than for the HTL + HDO configuration. Splitting the capital costs due to different process sections, we can observe that the costs due to the purification section are almost equal for both cases. The reaction section costs are higher for the HTL + HDO since it includes two reaction steps, and HTL is conducted at high pressure (larger than 110 bar). Further details regarding the effect of HDO pressure on the MSP of Alkylphenols are reported in Figure S4 of the Supporting Information. A pronounced difference is detected in the costs for the combined heat and energy production section: the cost for the direct HDO is nearly three times that for the HTL + HDO. This huge discrepancy is due to the need for several energy-generating sections in the direct HDO process, namely, two steam turbines and a gas turbine, while the HTL + HDO configuration requires only a gas turbine, as already reported in Section 3.1.

**Figure 6:**
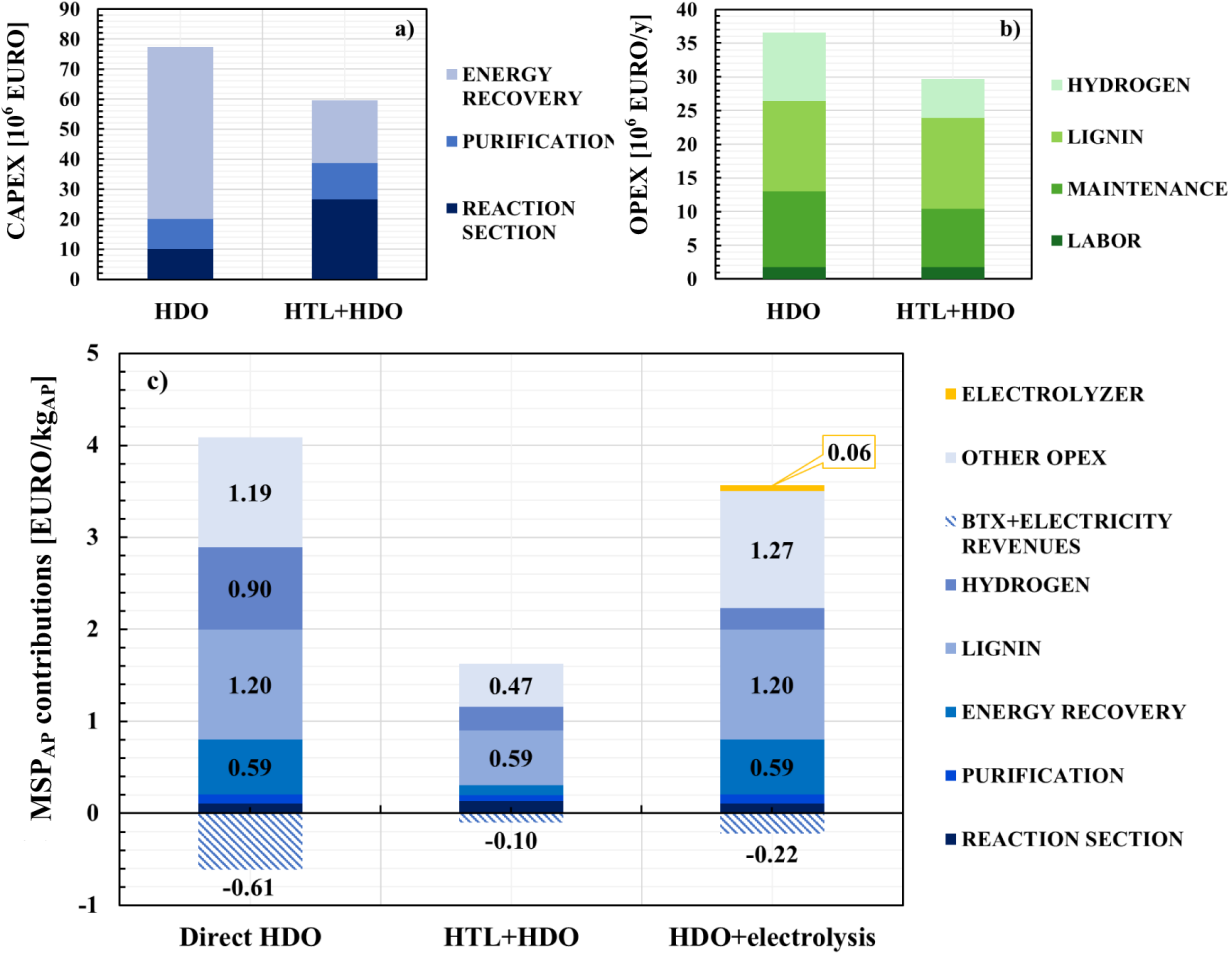
a) CAPEX, b) OPEX, and c) breakdown and contributions to the minimum selling price in the optimal cases.

A similar cost breakdown was carried out on the OPEX of the two processes (Figure 6b). Since the two plant configurations have the same lignin feed flow rate, the operating cost for the raw material is the same. However, the specific cost per unit mass of produced AP is much higher for direct HDO due to the lower overall yield to product. Moreover, the OPEX for the hydrogen supply is about 70% higher for the direct HDO process due to a higher H_2_ makeup demand, as previously stated. Maintenance costs are slightly higher for the direct HDO since they are assumed as proportional to the capital costs. On the whole, the total operating costs for direct HDO are about 25% higher than those for the HTL + HDO process.

Figure 6c reports the contribution of different terms on the overall alkylphenols MSP according to eq 2. Inspection of the histograms reveals that the purification and reaction sections have a very limited impact on MSP_AP_ for both configurations. Conversely, the contribution for raw materials is extremely significant for both processes: namely, lignin cost contributes around 35–40% in both cases, while hydrogen influence is higher for the direct HDO (around 26%) than for HTL + HDO (around 17%). The cost of the combined energy and heat production section is more remarkable in the direct HDO process reaching nearly 17% of the MSP_AP_ (around 0.60 EURO/kg_AP_), while the HTL + HDO does not exceed 7% of the total. It is also noteworthy to mention that, for the direct HDO, the revenues due to energy and BTX byproduct production appear non-negligible. In fact, because of the considerable amount of produced electric power, the obtained MSP_AP_ is reduced by around 0.61 EURO/kg_AP_, which, in turn, is compensated by the cost contribution for the energy recovery section (0.59 EURO/kg_AP_). On the whole, the difference between the MSPs computed for the two configurations seems to be due to the remarkably higher alkylphenol yield of the HTL + HDO process.

### 3.3 Economic Sensitivity Analysis

Cost analysis, reported in the previous section, reveals that raw materials cost has an astonishingly high impact on MSP_AP_ (approximately 55% for both lignin and hydrogen). For this reason, a sensitivity analysis was performed by varying the raw materials cost: hydrogen cost was varied between 2 and 6 EURO/kg, while lignin cost was varied between 0 and 400 EURO/t. The null cost of lignin corresponds to the case in which lignin is a byproduct or a waste stream in a biorefinery and is valorized on-site. The results are presented in Figure 7. For the direct HDO process, the lowest MSP_AP_ equal to 1.64 EURO/kg_AP_ was found for the best economic scenario, while the highest MSP_AP_ equal to 4.92 EURO/kg_AP_ was obtained for the upper limit costs of both lignin and hydrogen. It is remarkable that, even in the most favorable economic conditions, the lowest MSP_AP_ for the direct HDO process is still higher than the base case value for the HTL + HDO configuration. As far as the HTL + HDO configuration is concerned, MSP_AP_ varies in the range of 0.73–2.15 EURO/kg_AP_ applying the lowest and the highest cost values for the raw materials. Therefore, even the highest MSP_AP_ for the HTL + HDO process is lower than the base case MSP_AP_ for the direct HDO. Moreover, it is worth underlining that the effect of the cost of lignin is much more significant than that of hydrogen cost; MSP_AP_ is reduced by about 50% assuming lignin is free of charge, while it diminishes by less than 10% by halving the hydrogen cost.

**Figure 7:**
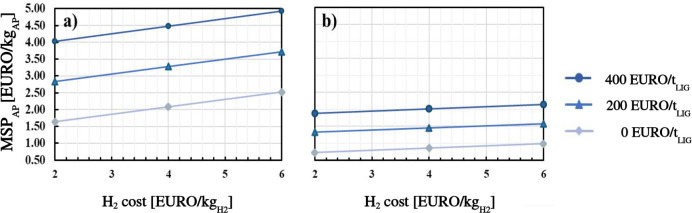
Effect of the raw material cost on the alkylphenols MSP for a) the direct HDO and b) the HTL + HDO process.

The electric power generated in the direct HDO process could be used to produce pure H_2_ in situ by employing an electrolyzer. Figure 6c reports the comparison between the MSP_AP_ for the HDO process with in situ electrolysis and the values for the previously analyzed cases. Exploiting electricity for in situ electrolysis allows the production of 73.7% of the hydrogen required by the process. This implies a decrease of MSP_AP_ from 3.28 to 3.14 EURO/kg_AP_ for the direct HDO process, since the savings on the H_2_ purchase cost overcome the additional investment for the electrolyzer and the lost revenues due to the missed electricity sales, as it can be seen from the cost breakdown reported in Figure 6c. Nevertheless, the HTL + HDO configuration remains the most convenient process solution.

## 4 Conclusions

This study provided a rigorous techno-economic assessment comparing two distinct pathways for converting lignin into high-value chemicals: direct HydroDeOxygenation (HDO) and the sequential HydroThermal Liquefaction followed by HydroDeOxygenation (HTL + HDO). The results decisively establish the HTL + HDO configuration as the superior route for the coproduction of alkylphenols (AP) and aromatic hydrocarbons (BTX), demonstrating strong technical performance and clear economic advantages.

The HTL pretreatment step significantly enhanced the efficiency of the lignin depolymerization. Furthermore, the HTL + HDO configuration achieves alkylphenol yields of up to 35% by weight (on an inlet lignin basis), which is two to three times greater than the yield achievable through direct HDO. This high yield is attained because the HTL stage handles the initial depolymerization almost entirely, generating the reactive lignin intermediate LD1. Subsequently, the hydrogen reacting in the HDO stage is primarily tasked with biocrude upgrading rather than the initial solid lignin breakdown.

This optimized reaction mechanism translates directly to lower operating requirements. HTL + HDO demands more than 40% less fresh hydrogen overall (ranging from 20 to 27 kg/t lignin), compared to the direct HDO route (26–40 kg/t lignin). Optimal HTL conditions, maximizing the production of LD1, were identified at a residence time between 40 and 50 min and the lowest possible inlet temperature. Furthermore, both process configurations proved to be thermally self-sufficient, eliminating the need for external hot utilities.

The combined technical benefits result in a dramatic reduction in the production costs. Direct HDO CAPEX is 30% higher than HTL + HDO. Notably, the cost of the combined heat and power section for HDO is nearly three times higher due to its need for a more complex energy recovery system involving two steam turbines and one gas turbine, whereas HTL + HDO requires only one gas turbine. Total OPEX for direct HDO is about 25% higher. Specifically, the OPEX for hydrogen supply is about 70% higher in direct HDO due to the increased H_2_ makeup demand. The resulting Minimum Selling Price for alkylphenols via HTL + HDO is 1.52 EURO/kgAP, which is less than half of the optimal MSP of the direct HDO process (3.47 EURO/kgAP). The HTL + HDO configuration’s lower MSP places it below the reported market prices for AP (2.51–3.14 EURO/kg), confirming its substantial profit opportunities.

A comprehensive sensitivity analysis was also conducted to assess the impact of market fluctuations, varying the cost of lignin and hydrogen, including the option of in situ hydrogen production through electrolysis. This analysis demonstrated that the lignin market price has the greatest impact on the minimum selling price of alkylphenols, resulting in variations of more than 1 EURO/kg_AP_. Even under the most favorable economic conditions for direct HDO (low hydrogen cost and free lignin), the HTL + HDO configuration remained the most economically convenient process solution. The results affirm that coupling the HTL with HDO maximizes product yields and minimizes costs, securing profitability for lignin valorization.

## Supplementary Material



## Notes

SupplementarymaterialGiuianoetal2025.pdf reporting the following additional contents: Lignin properties; Details on Reactors’ Modeling; Details on the Economic Analysis; HDO and HTL process conditions optimization; Details on the distillation column design; Details on heat integration; Details on CAPEX results (PDF)

## §

Cannon Artes S.p.A. Via Irno, 221, 84135 Salerno (SA), Italy

## Abbreviations

AF: annualization factor
AP: Alkylphenols
BTX: benzene-toluene-xylene
CAPEX: capital expenditure
CC: annualized capital costs
CCS: carbon capture and storage
CF_*j*_: cash flow for year *j*
DP: depreciation
*f*_*j*_: fraction of the TIC spent during year
H/C: hydrogen-to-carbon atomic ratio
HDO: Hydrodeoxygenation
HHV: higher heating value
HTL: hydrothermal liquefaction
HyThUp: hydrothermal upgrading
*i*: interest rate
*L*: reactor length
MOAMON: mixed oxygenated aromatic monomers
MSP: minimum selling price
MSP_AP_: alkylphenols minumum selling price
*n*: expected number of years of the plant
NPV: net present value
NREL: National Renewable Energy Laboratory
OPEX: operating expense
PSA: pressure swing adsorption
Rev_*j*_: revenues of year *j*
*t*: tax rate
TAC_*j*_: total annual operating cost for year *j*
TIC: total capital investment cost
Tin: reactor inlet temperature
*W*/*L*: water-to-lignin mass ratio
WC: working capital
