# Supplementary material for: Techno-Economic Assessment of Two Process Routes for Lignin-Derived Alkylphenols and Aromatic Hydrocarbons
Source: ACS Omega. 2026 Feb 6;11(6):9201–11. doi: 10.1021/acsomega.5c08334 (PMC12917615; doi:10.1021/acsomega.5c08334)
Supplement: Supplementary file 1 [file ao5c08334_si_001.pdf]

Supplementary Material of the paper

Techno-economic assessment of two process routes for lignin-derived alkylphenols and aromatic hydrocarbons

Aristide Giuliano[a], Aniello Di Giacomo[b], Isabella De Bari[a], Diego Barletta[b*]

[a]ENEA, Italian National Agency for New Technologies, Energy and Sustainable Economic Development, S.S. 106 Ionica, Laboratory of Technologies and Processes for Biorefineries and Green Chemistry, km 419+500, Rotondella (MT), Italy

[b]Department of Industrial Engineering, University of Salerno, Via Giovanni Paolo II 132, I-84084 Fisciano (SA), Italy

* corresponding author, Email: dbarletta@unisa.it

CONTENTS

Lignin properties

Details on Reactors' Modelling

Details on the Economic Analysis

HDO and HTL process conditions optimization

Details on the distillation column design

Details on heat integration

Details on CAPEX results

Lignin properties

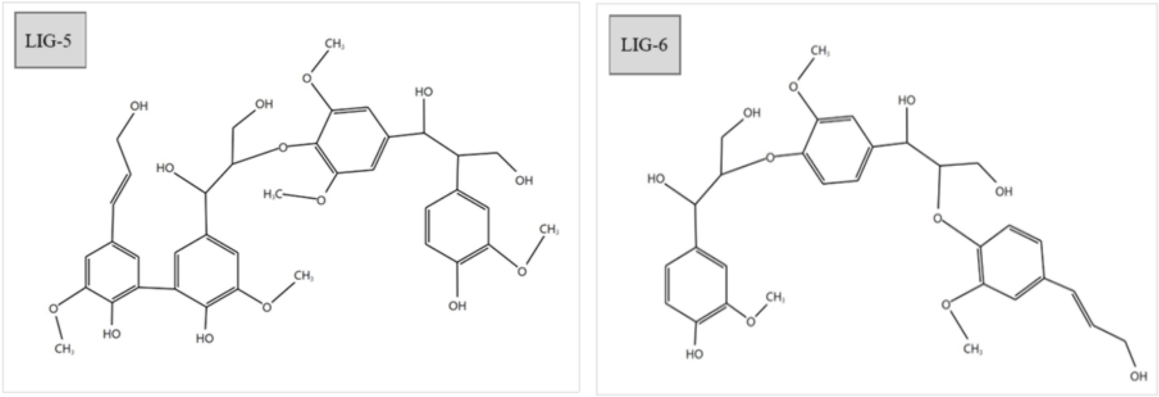

Figure S1. Molecular structure of LIG-5 and LIG-6 lignin oligomers.

Table S1. Lignin oligomers properties

**Property**	**LIG-5**	**LIG-6**	**Units**	**Reference**
**Formula**	$C_{38}H_{44}O_{14}$	$C_{30}H_{36}O_{11}$	-	a)
**MW**	724.76	572.61	kg/kmol	a)
**MR (H/G/S)**	(0/3/1)	(0/3/0)	-	a)
**BR (β-O-4/β-1/5-5)**	(1/1/1)	(2/0/0)	-	a)
**DHSFRM**	-1.97E+09	-1.59E+09	J/kmol	b)
**DGSFRM**	-2.36E+09	-1.86E+09	J/kmol	b)
**HHV**	-23.52	-23.57	MJ/kg	c)

MR ratio of structural units: p-hydroxyphenyl (H), guaiacyl (G), and syringyl (S), BR bond ratio, DHSFRM Solid enthalpy of formation, DGSFRM Solid standard Gibbs free energy, HHV High Heating Value

[a] Bertarelli P.A. Simulations of the Lignin Hydrogenation Process, Master Thesis in Chemical Engineering, University of Salerno, 2022

[b] Azad, T., Torres, H.F., Auad, M.L., Elder, T., Adamczyk, A.J. Isolating Key Reaction Energetics and Thermodynamic Properties during Hardwood Model Lignin Pyrolysis. Phys. Chem. Chem. Phys. 2021, 23 (37), 20919–20935. https://doi.org/10.1039/D1CP02917G.

[c] Voitkevich, O., Kabo, G., Blokhin, A., Paulechka, Y., Shishonok, M. Thermodynamic Properties of Plant Biomass Components. Heat Capacity, Combustion Energy, and Gasification Equilibria of Lignin, Journal of Chemical & Engineering Data, 2012, 57, 1903−1909. https://doi.org/10.1021/je2012814

Table S2. Temperature-dependent parameters for the considered oligomers

**Property**	**C1**	**C2**	**Tmin**	**Tmax**	**Units**	**Reference**
**CPSPO**	31431.7	394.427	298.15	1000	J/mol K	d)
**VSPOLY**	0.0817	0	1000	1000	m^3/kmol	d)

Solid Heat Capacity (CPSPO) = C1+C2·T

Solid specific Volume (VSPOLY) = C1

[d] Wooley, R.J., Putsche, V. Development of an ASPEN PLUS physical property database for biofuels components. No. NREL/TP-425-20685. National Renewable Energy Lab. (NREL), Golden, CO (USA), 1996.

Details on Reactors' Modelling

Hydrodeoxygenation reactions

The reactions considered to model the Hydrodeoxygenation reactor with a thermodynamic-based temperature approach are listed in Table . The ΔT values (the temperature shift with respect to the reaction temperature of 350°C), obtained by a trial-and-error method, and the sign of the standard enthalpy change are reported in Table S3.

The chemical reaction system is modelled by a REQUIL block in Aspen Plus.

Table S3. Considered reactions to model the HDO step using the temperature approach.

#	reaction		ΔHr°	ΔT [°C]
**1**	PHENOL + ETHANOL $\rightarrow$ P-HYDROXYBENZALDEHYDE + H_2 + CH_4	> 0	Endothermic	0
**2**	PHENOL + H_2 $\rightarrow$BENZENE + WATER	< 0	Exothermic	3800
**3**	BENZENE + 3 H_2 $\rightarrow$CYCLOHEXANE	< 0	Exothermic	185
**4**	GUAIACOL + ETHANOL $\rightarrow$$CH_4$ + VANILLIN + H_2	> 0	Endothermic	200
**5**	ANISOLE + H_2 $\rightarrow$TOLUENE + WATER	< 0	Exothermic	150
**6**	TOLUENE + 3 H_2 $\rightarrow$METHYL CYCLOHEXANE	< 0	Exothermic	200
**7**	SYRINGOL + ETHANOL $\rightarrow$$CH_4$ + SYRINGALDEHYDE + H_2	> 0	Endothermic	0
**8**	SYRINGOL + $H_2$$\rightarrow$DIMETHOXYBENZENE + WATER	< 0	Exothermic	1300
**9**	DIMETHOXYBENZENE + 2 H_2 $\rightarrow$XYLENE + 2 WATER	< 0	Exothermic	1100
**10**	XYLENE + 3 H_2 $\rightarrow$DIMETHYL CYCLOHEXANE	< 0	Exothermic	150
**11**	GUAIACOL + H_2 $\rightarrow$ANISOLE + WATER	< 0	Exothermic	0
**12**	XYLENE + 2 H_2 $\rightarrow$BENZENE + 2 CH_4	< 0	Exothermic	3400
**13**	PHENOL + 3 H_2 $\rightarrow$CYCLOHEXANOL	< 0	Exothermic	50
**14**	GUAIACOL + H_2 $\rightarrow$CATECHOL + CH_4	< 0	Exothermic	-250
**15**	TOLUENE + H_2 $\rightarrow$BENZENE + CH_4	< 0	Exothermic	4800
**16**	ETHANOL + 2 H_2 $\rightarrow$2 CH_4 + WATER	< 0	Exothermic	0
**17**	PROPENOL + 2 H_2 $\rightarrow$ETHANOL + CH_4	< 0	Exothermic	200
**18**	ETHANOL + WATER $\rightarrow$ACETIC ACID + 2 H_2	> 0	Endothermic	0
**19**	WATER $\rightarrow$$H_2$ + 0.5 O_2	> 0	Endothermic	0
**20**	CH_4 + 1.5 O_2 $\rightarrow$CO + 2 H_2O	< 0	Exothermic	0
**21**	GUAIACOL + H_2 $\rightarrow$CRESOL + WATER	< 0	Exothermic	-243
**22**	CO + 0.5 O_2 $\rightarrow$$CO_2$	< 0	Exothermic	0
**23**	CYCLOHEXANE + H_2 $\rightarrow$N-HEXANE	< 0	Exothermic	4000
**24**	BENZENE + 9 H_2 $\rightarrow$6 CH_4	< 0	Exothermic	2670
**25**	FORMIC ACID + 3 H_2 $\rightarrow$$CH_4$ + 2 WATER	< 0	Exothermic	-100

Hydrothermal liquefaction reactions

The kinetic scheme used to model the hydrothermal liquefaction reactor is summarized in Figure . The lumped model was adapted from (Forchheim, et al. 2014) The first order kinetics rate constants and relative activation energies are reported in Table .

The HTL tubular adiabatic reactor was modelled by a RPLUG block in Aspen Plus.

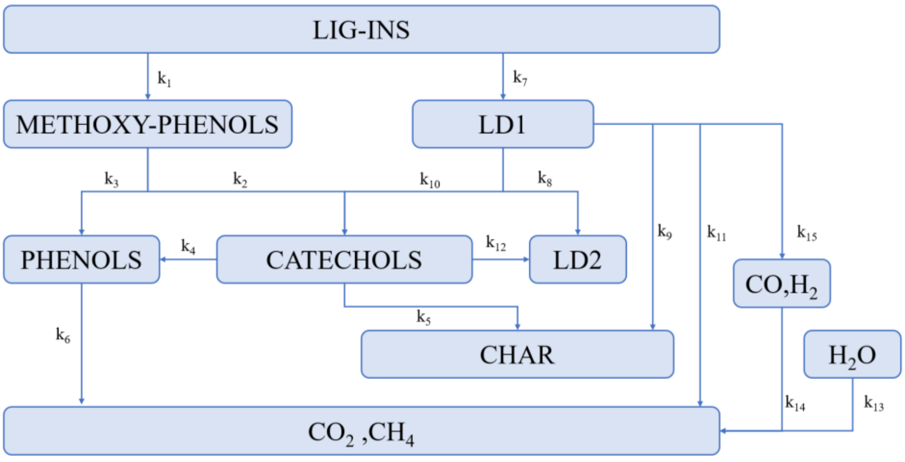

Figure S2. HTL lumped reaction scheme, adapted from Forchheim et al.

Table S4. Reactions, kinetic constants and activation energies of the HTL first order kinetic model by Forchheim et al. (2014)

#	reaction	k_0 [1/s]	E_{ATT} [kJ/mol]
**1**	LIG-INS + a H_2O → b PHENOL + c GUAIACOL + d SYRINGOL + e CO_2 + f H_2	0.41	58
**2.1**	GUAIACOL + H_2 → CATECHOL + CH_4	34841.56	101
**2.2**	SYRINGOL + H_2 → METHOXY-CATECHOL + CH_4	34841.56	101
**3.1**	GUAIACOL → PHENOL + 0.5 CO_2 + 0.5 CH_4	1639.41	94
**3.2**	SYRINGOL → PHENOL + CO_2 + CH_4	1639.41	94
**4.1**	CATECHOL + CO → PHENOL + CO_2	0.16	55
**4.2**	METHOXY-CATECHOL + 3.667 CO → 1.333 PHENOL + 2.667 CO_2	0.16	55
**5**	negligible	0.00	3037
**6**	PHENOL + 3 H_2O + 2 H_2 → 2 CO_2 + 4 CH_4	0.20	66
**7**	LIG-INS → LIG-LD1	0.26	32
**8**	LIG-LD1 → LIG-LD2	798.24	81
**9**	LIG-LD1→ g C + h H_2O + i H_2	6.20	58
**10**	LIG-LD1 + j H_2O → k PHENOL + l CATECHOL + m METHOXYCATECHOL + n CO_2 + o H_2 + p CH_4	5.67	72
**11**	LIG-LD1 → q CO_2 + r CH_4 + s C	98.18	75
**12**	negligible	0.00	296
**13**	H_2O + CO → CO_2 + H_2	0.54	48
**14**	CO + H_2 → 0.5 CO_2 + 0.5 CH_4	2.27	42
**15**	LIG- LD1 → t H_2 + u CO + v C	9.52	69

Details on the Economic analysis

Table S5. CAPEX estimation parameters for the power law cost correlations, actualized to 2023.

Equipment/ process step	Capacity measure	Units	Base size	Base cost [EURO]	Cost exp.	Installation factor
Distillation Column[g]	Diameter<br>Height	m<br>m	0.91<br>1.22	7470	1.05<br>0.81	24
Distillation Tray[g]	Diameter<br>Height	m<br>m	0.61	1344	1.45<br>0.97	24
Heat exchanger*,[g]	Area	m^2	0.51	1882	0.024	2.33
Heat exchanger**,[g]	Area	m^2	37.2	31372	0.65	2.29
Flash unit#,[g]	Diameter<br>Height	m<br>m	0.91<br>1.22	8130	1.05<br>0.81	5
Decanter##,[g]	Volume	m^3	6	20300	0.82	6.25
PSA[e]	Bed size	m^3	27	$3.00 \cdot 10^6$	1	1
Steam generation section[h]	Steam flow	kg/s	39.2	$1.48 \cdot 10^7$	0.6	1.84
Steam turbine[h]	Power	MW	10.3	$1.08 \cdot 10^7$	0.7	1.86
Gas turbine[h]	Power	MW	26.3	$3.42 \cdot 10^7$	0.7	1.86
Steam boiler[h]	Power	MW	173	$5.48 \cdot 10^7$	0.73	1
Solid separation section[h]	Solid flow	tonne/h	10.1	$2.12 \cdot 10^6$	0.65	2.2
Pump[i]	Power	kWe	4	20300	0.55	9
Compressor[i]	Power	kWe	250	203000	0.46	16.187
Agitated reactor×,[i]	Volume	m^3	1	21789	0.45	1
Electrolyzer[f]	Power	kW	1	500	1	1
HTL reactor[l]	Volume	m^3	4.74	390934	1	2

*for 0.185<area<9.29 m^2; ** for area>9.29 m^2; # 3.16 m/s liquid velocity considered; ## 1h residence time is considered

× It is noteworthy to highlight that the power law was not directly applied to the overall required volume of the HDO agitated reactor, which resulted of the order of a thousand cubic meter. In fact, a set of numerous reactors with a 50 m3 volume was assumed and the cost correlation was applied to each of them accordingly.

e) Giuliano, A.; Poletto, M.; Barletta, D. Pure Hydrogen Co-Production by Membrane Technology in an IGCC Power Plant with Carbon Capture. International Journal of Hydrogen Energy 2018, 43 (41), 19279–19292. https://doi.org/10.1016/j.ijhydene.2018.08.112.
f) Reuß, M.; Grube, T.; Robinius, M.; Preuster, P.; Wasserscheid, P.; Stolten, D. Seasonal Storage and Alternative Carriers: A Flexible Hydrogen Supply Chain Model. *Applied Energy* **2017**, *200*, 290–302. https://doi.org/10.1016/j.apenergy.2017.05.050.
g) Biegler, L. T.; Westerberg, A. W.; Grossmann, I. E. *Systematic Methods of Chemical Process Design*; Prentice-Hall, 1997.
h) Hamelinck, C. N.; Van Hooijdonk, G.; Faaij, a. P. C. Ethanol from Lignocellulosic Biomass: Techno-Economic Performance in Short-, Middle- and Long-Term. *Biomass and Bioenergy* **2005**, *28* (4), 384–410. https://doi.org/10.1016/j.biombioe.2004.09.002.
i) Smith, R. *Chemical Process Design and Integration,* 2nd Edition, 2005, Wiley.
l) Knorr, D.; Lukas, J.; Schoen, P. Production of Advanced Biofuels via Liquefaction Hydrothermal Liquefaction Reactor Design. *OurEnergyPolicy* **2013**. https://doi.org/10.2172/1111191.

Table S6. Assumptions used for OPEX calculation.

	value	Units
operating hours/year	7200	h/year
plant operating years	20	Years
Operators	20	-
BTX price	0.5	EURO/kg
electric energy price	50	EURO/MWh
lignin cost	0.2	EURO/kg
hydrogen cost	4	EURO/kg
HDO catalysts consumption	5	wt% of the inlet dry lignin
HDO catalyst cost (S-NiMo/MgO-La_2O_3)	100	EURO/kg

HDO and HTL process conditions optimization

After defining and validating the kinetic model in the process simulation, the hydrothermal liquefaction reactor was studied to understand the yield (with respect to the lignin inlet mass flow) of the main HTL outputs, namely biocrude, gas phase and unreacted solid. The aqueous phase was not considered, since it is always a very diluted stream, containing a negligible quantity of organic compounds. The results of the simulations are reported in Figure S3.

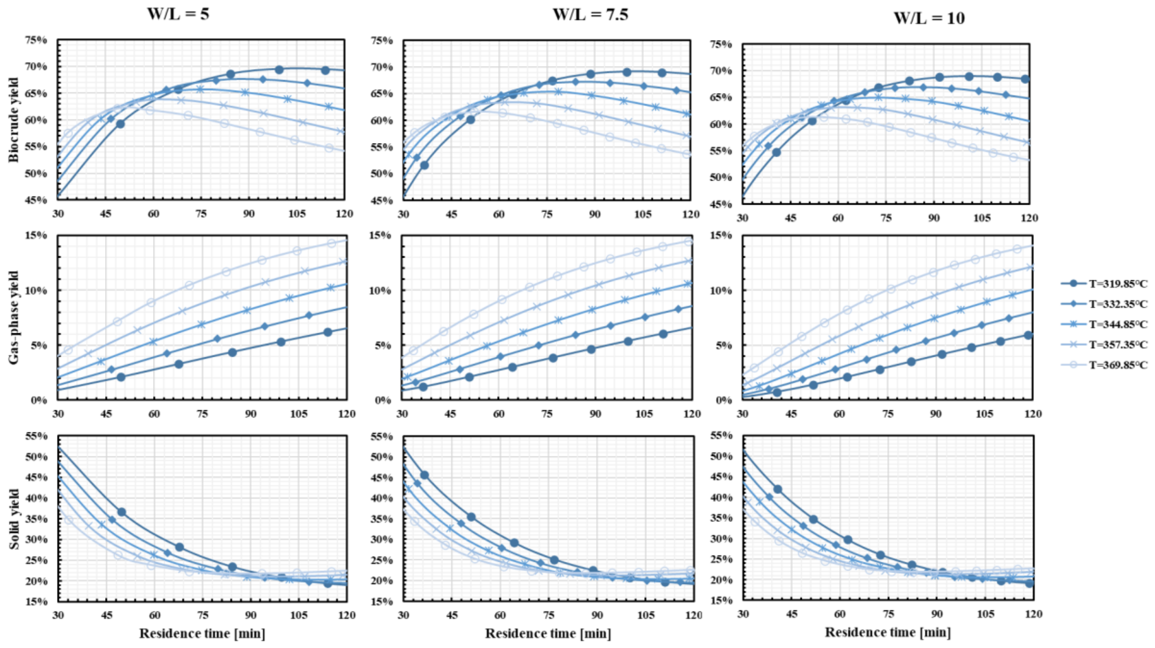

Figure S3. Biocrude, gas phase and unreacted solid yield via HTL at different temperatures and water/lignin (W/L) ratios.

The degrees of freedom of both reactors investigated in order to minimize the alkylphenols minimum selling price and the relevant range of variation are summarized in the following tables.

Table S7. Investigated conditions for the direct HDO hydrogen feed.

#	feed H/C atomic ratio	inlet H_2 mole flow [kmol/h]	inlet H_2 mass flow [kg/h]
**1**	4	980	1975.56
**2**	5	1225	2469.45
**3**	6	1470	2963.34

Table S8. Investigated pressures for the HDO reactor.

#	HDO pressure [bar]
**1**	50.00
**2**	55.55
**3**	61.11
**4**	66.67
**5**	72.22
**6**	77.78
**7**	83.33
**8**	88.89
**9**	94.44
**10**	100.00

Table S9. Investigated length values for the HTL reactor.

#	L [m]
**1**	0.50
**2**	4.00
**3**	7.50
**4**	11.00
**5**	14.50
**6**	18.00
**7**	21.50
**8**	25.00
**9**	28.50
**10**	32.00

Table S10. Investigated inlet temperature values for the HTL reactor.

#	T_{in} [°C]
**1**	319.85
**2**	332.35
**3**	344.85
**4**	357.35
**5**	369.85

Table S11. Investigated feed water/lignin ratios and H_2O inlet flows for the HTL reactor.

#	*W/L* [-]	water mass flow [kg/h]
**1**	5	46750
**2**	7.5	70125
**3**	10	93500

Effect of HDO pressure on the Minimum Selling Price of Alkylphenols (MSP_{AP})

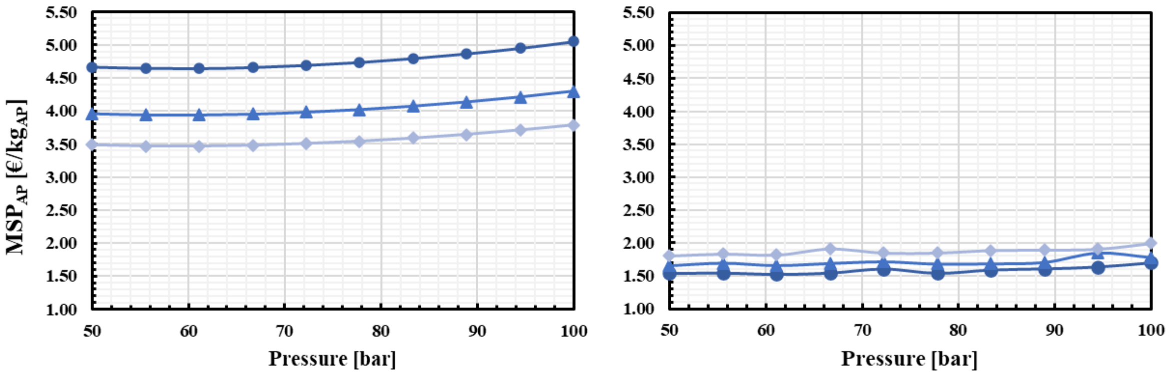

Figure S14. Alkylphenols MSP for direct HDO (left) and HDO+HTL (right) for H/C= 4 (circles); 5 (triangles); 6 (diamonds).

Details on the distillation column design

The distillation units were modeled and sized by rigorous stage-by-stage methods (RADFRAC block)

Table S12. Distillation columns performance and design results for both direct HDO and HTL+HDO.

	COL1, HDO	COL2, HDO	COL1, HTL+HDO	COL2, HTL+HDO	units
**Number of stages**	10	6	10	6	-
**Reflux ratio**	0.9	3	0.9	3	-
**Height**	10.97	7.32	10.97	7.32	m
**Diameter**	0.91	0.46	1.07	0.46	m
**AP purity**	98.4%	-	98.2%	-	-
**AP recovery**	98.2%	-	95.6%	-	-
**BTX purity**	-	99.1%	-	95.1%	-
**BTX recovery**	-	98.9%	-	99%	-

Details on heat integration

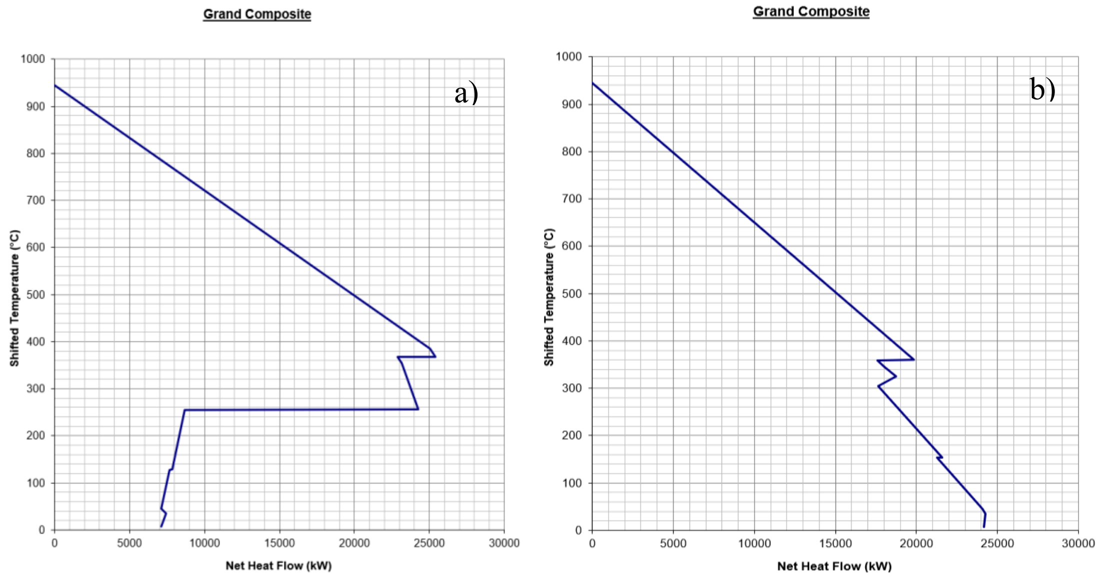

Figure S5. Heat integration Grand Composite Curves for direct HDO (a) and HDO+HTL (b).

Details on CAPEX results

Table S13. CAPEX for the economic optimal cases considered.

DIRECT HDO			HTL + HDO	
**equipment**	**bare modulus cost [EURO]**		**equipment**	**bare modulus cost [EURO]**
**HEAT EXCHANGER NETWORK**	526,000		**HEAT EXCHANGER NETWORK**	368,000
**HDO reactor**	9,887,000		**HDO reactor**	3,196,000
**CATALYST**	187,000		**HTL reactor**	21,579,000
**FLASH1**	348,000		**CATALYST**	133,000
**FLASH2**	358,000		**FLASH1**	143,000
**FLASH3**	334,000		**FLASH2**	147,000
**SOLID SEPARATION**	3,112,000		**FLASH3**	119,000
**PSA**	321,000		**SOLID SEPARATION**	2,967,000
**DIST. COL. 1**	1,266,000		**PSA**	108,000
**DIST. COL. 2**	420,000		**DECANTER**	638,000
**WATER PUMP 1**	504,000		**DIST. COL. 1**	1,509,000
**WATER PUMP 2**	847,000		**DIST. COL. 2**	423,000
**H2 MAKEUP COMPRESSOR**	2,507,000		**WATER PUMP**	1,496,000
**H2 RECYCLE COMPRESSOR**	1,373,000		**BIOCRUDE PUMP**	75,000
**HRSG-SOLID**	12,339,000		**H2 MAKEUP COMPRESSOR**	5,435,000
**HRSG-GAS**	6,457,000		**H2 RECYCLE COMPRESSOR**	824,000
**STEAM TURBINES**	23,275,000		**SOLID COMBUSTOR**	13,294,000
**GAS TURBINE**	13,341,000		**GAS TURBINE**	7,167,000
**CTBM**	77,402,000		**CTBM**	59,621,000
**CTDC**	108,363,000		**CTDC**	83,470,000
**CTCI**	112,698,000		**CTCI**	86,809,000
